# Evidence-based guidelines for physiotherapy management of patients with multiple myeloma: study protocol

**DOI:** 10.1186/s13643-018-0785-7

**Published:** 2018-08-16

**Authors:** Deepa Jeevanantham, Venkadesan Rajendran, Line Tremblay, Céline Larivière, Andrew Knight

**Affiliations:** 1 Medicine, Oncology and Palliative Care, Health Sciences North, Sudbury, Ontario Canada; 2 Northern Ontario School of Medicine, Sudbury, Ontario Canada; 3 Faculty of Health, School of Human Kinetics, Laurentian University, Sudbury, Ontario Canada

**Keywords:** Multiple myeloma, Physiotherapy, Guidelines

## Abstract

**Background:**

Patients with multiple myeloma (MM) are often treated with chemotherapy, radiation, and, if indicated, autologous stem cell transplant. In addition to side effects of the treatment, patients with MM often have bone pain, pathological fractures, spinal cord compressions, fatigue, and muscle weakness, which negatively impact functional performance and quality of life. Currently, there are no related guidelines for safe and effective physiotherapy (PT) management. Accordingly, the aim of the present study is to develop guidelines for effective physiotherapy management of patients with MM by systematically reviewing and evaluating the available evidence followed by a consensus process to specifically describe the research questions as detailed below.

**Methods/design:**

Physiotherapy management guidelines for patients with multiple myeloma will be developed based on the results of a systematic search of the following databases: US National Library of Medicine Database (PubMed), Medical Literature Analysis and Retrieval System Online (MEDLINE), Excerpta Medica Database (EMBASE), Cumulative Index to Nursing and Allied Health Literature (CINAHL), Elton B. Stephens Co. (EBSCO), Web of Science, Database of Abstracts of Reviews of Effects (DARE), Cochrane Database of Systematic Review, and Physiotherapy Evidence Database (PEDro). All articles will be screened for inclusion and exclusion criteria. Relevant potential articles will be identified and systematically reviewed for final phase of inclusion. Two independent reviewers will systematically review and analyze the quality of identified articles using standardized assessment tools. Scientific conclusions will be drawn and recommendations will be made based on a critical appraisal process. The guideline development will also be based on the team’s judgment about the overall quality of the studies and a consensus process.

**Discussion:**

Draft guidelines will be developed in the form of action statements based on the strength of evidence and grades of recommendations. The draft guidelines will be reviewed internally by two independent reviewers using AGREE II and externally by a methodological expert from Evidence-Based Care – Cancer Care Ontario and will be sent to the Canadian Physiotherapy Association (CPA) for feedback from physiotherapists.

**Systematic review registration:**

PROSPERO CRD42017064056

**Electronic supplementary material:**

The online version of this article (10.1186/s13643-018-0785-7) contains supplementary material, which is available to authorized users.

## Background

Multiple myeloma (MM) is a malignancy of plasma cells that normally reside in bone marrow, producing immunoglobulins (antibodies). In MM, the uncontrolled growth of these cells result in increased osteoclastic activity with the destruction of bone matrix leading to hypercalcemia and an increased risk of fracture [1]. According to the 2017 Canadian Cancer Statistics report, MM accounts for 1.6% of all new cases of cancer in men and 1.2% of all new cases of cancer in women [2]. The incidence rate of MM is three times higher in more developed countries compared to lesser-developed countries [3]. MM is typically diagnosed at the median age of 69 years, and the median survival rate is 3 to 4 years [4]. Patients with MM may present with hypercalcemia, renal insufficiency, anemia, and bone disease [5]. The bone tissue destruction in MM results in bone pain, pathological fractures, and less frequently spinal cord compression.

Patients with MM are generally treated with chemotherapy, radiation and potentially autologous stem cell transplant, and/or radiation. High-dose chemotherapy is a systemic therapy that uses high doses of cytotoxic drugs to destroy myeloma cells. In addition to destroying myeloma cells, the cytotoxic drugs also destroy hematopoietic stem cells leading to neutropenia and thrombocytopenia [6]. This puts patients at the risk of developing infections and hemorrhage. Typically, patients receiving high-dose chemotherapy always undergo stem cell re-infusion to restore marrow function [6]. In addition, patients receiving high-dose chemotherapy may experience numerous side effects including, but not limited to, nausea, vomiting, diarrhea, stomach pain, heart burn, mouth sores, hair loss, and skin rash [6]. These patients tend to be in bed for prolonged periods of time.

Radiation therapy is often prescribed for management of bone pain and osteolytic lesions, preventing paralysis in patients with spinal cord compression. Radiation therapies induce apoptosis and reduce the tumor size [7]. This results in an analgesic effect and may prevent paralysis by decompressing the nerves [8]. In addition, radiation therapy also induces re-calcification and reduces the risk of fractures [8]. Patients with cancer including multiple myeloma, often experience fatigue due to both cancer and cancer related treatments. Cancer-related fatigue is defined as “a distressing, persistent, subjective sense of physical, emotional, and/or cognitive tiredness or exhaustion related to cancer or cancer treatment that is not proportional to recent activity and interferes with usual functioning” ([9], Pg.1). It is estimated that 30 to 91% of patients receiving chemotherapy and 25 to 83% of patients receiving radiation therapy experience cancer-related fatigue [2, 10]. Cancer-related fatigue is not relieved by rest or sleep, and it significantly affects functional performance and quality of life [11].

Patients considered as candidates for high-dose chemotherapy and autologous transplantation are admitted to hospital for a 2- to 3-week period. During the in-patient stay, number of factors may significantly affect the physical performance and functional status of hospitalized patients with MM including, prolonged periods of bed rest with muscle inactivity, older age, effects of cancer treatments including fatigue. Careful attention to pain management, safe mobilization by providing exercises that are safe and preventing functional decline (deconditioning), improving strength and physical performance are significant in improving quality of life and also help in preventing complications.

Physiotherapy (PT) is a part of cancer rehabilitation and is often recommended for patients with MM; however, there are no specific guidelines for safe and effective PT management. Evidence-based guidelines also known as clinical guidelines are systematically developed statements that guide health care practitioners and patients in making decisions on the selection of appropriate health care services/treatments [12–14]. Guidelines are typically developed using systematic reviews by identifying evidence supporting clinical questions, critically appraising and grading the quality of the evidence based on methodological rigors, and providing recommendations. Evidence-based guidelines assist with incorporating best evidence into practice [13–15]. However, it is important to remember that treatment plans and decisions should be made based on individual clinical circumstances [14].

The aim of the present study is to develop guidelines for effective physiotherapy management of patients with MM by systematically reviewing and evaluating the available evidence followed by an expert review process to specifically describe the research questions as detailed below.

## Methods

The following steps recommended by the American Physical Therapy Association (APTA) [16] will be followed for drafting recommendations and determining levels of evidence: (1) determine research questions, (2) conduct a systematic literature search, (3) critically appraise evidence and extract data, and (4) draft recommendations and determine levels of evidence. Recommendations for research questions with inadequate or lack of evidence will be developed based on a consensus process. A similar method (i.e., consensus-based recommendation) has been used by other groups to develop guidelines in the absence of high-quality evidence [17].

The APTA recommends draft guideline review by content experts and stakeholders ([16], page 36). Accordingly, the recommendations will be revised based on feedback and consensus from experts through a consensus process. The recommendations will be sent to the CPA to obtain feedback from physiotherapists. The guidelines will also be reviewed by a methodological expert from Evidence-Based Care - Cancer Care Ontario.

### Development of research questions

Defining the research questions is a critical step that provides the framework for defining eligibility criteria and facilitating good clinical research [14, 15]. This study will address the following answerable research questions developed based on the PICO (population, intervention, control, and outcomes) format [18]. Table 1 shows the key research questions in PICO format. Accordingly, the specific objectives of the present study is to develop evidence-based guidelines for the following: (1) safe mobilization of patients with MM in an acute care setting, (2) effective pain management in patients with MM, (3) physiotherapy (PT) management in patients with MM receiving chemotherapy, (4) PT management in patients with MM receiving radiation, (5) PT management in patients with MM receiving a stem cell transplantation, and (6) management of fatigue and muscle weakness in patients with MM.

**Table 1:** PICO (population, intervention, control, and outcomes)

Review question	What are the available evidence for the following: - Safely mobilizing patients with multiple myeloma in an acute care setting - Effective pain management in patients with multiple myeloma - Physiotherapy (PT) management in patients with multiple myeloma receiving chemotherapy - PT management in patients with multiple myeloma receiving radiation - PT management in patients with multiple myeloma receiving a stem cell transplantation - Management of fatigue and muscle weakness in patients with multiple myeloma
Population	Multiple myeloma or myeloma or plasma cell myeloma
Intervention	Physiotherapy or physical therapy, ambulation, mobilization, mobility, functional mobility training, exercise, strength training, endurance training, cardio-pulmonary exercise, aerobics exercise, anaerobic exercise, high-intensity exercise, low-intensity exercise, home-based exercise, multi-modal exercise, flexibility exercise, physical activity, pain management, and fatigue management
Comparator	Stated interventions compared with placebo or no interventions
Outcomes	All possible outcomes

### Study registration and design

This study is registered in the International Prospective Register of Ongoing Systematic Reviews (PROSPERO) published by Centre for Reviews and Dissemination, University of York, in order to reduce duplication of effort and publication bias [19, 20]. This study will adhere to the Preferred Reporting Items for Systematic Reviews and Meta-analyses Protocol (PRISMA – P 2015) checklist. The PRISMA – P 2015 checklist is comprised of 17 items, categorized into administrative information, introduction, and methods [21, 22]. The completed PRISMA-P checklist is included as an additional file (see Additional file 1).

### Types of studies

The studies that are of interest for this current investigation are those that focus on physical therapy intervention in patients with MM who had been actively receiving cancer treatment and in follow-up care. However, a preliminary literature search suggested that few randomized controlled trials addressing this topic were available. Therefore, the guideline development team has decided to include systematic reviews, meta-analyses, interventional and observational studies, and case series, which collectively might be informative and helpful in developing the guidelines. In addition, a gray literature search will be conducted to ensure that any potential informative articles are not missed. Only articles published in the English language from the earliest to Aug 2017 will be included.

### Types of participants

Articles with human participants diagnosed with MM of any age, regardless of gender, tumor stage, and type of cancer treatment will be included.

### Types of interventions/exposure and comparison

Articles examining the effects of exposure or the effects of physical therapy interventions including physical activity, exercise, and other modalities will be included. Articles with comparison groups with standard interventions or no intervention will be included.

### Types of outcome measures

Articles reporting on the following primary outcomes of interest will be included: (1) pain, (2) safety, (3) laboratory values, (4) length of stay, (5) range of motion, (6) muscle strength, (7) balance, (8) gait, (9) endurance, (10) fatigue, (11) physical performance, (12) functional performance, and (13) aerobic capacity. The secondary outcomes of interest are (1) quality of life and (2) anxiety/depression.

### Search strategy

A detailed literature search will be conducted on the US National Library of Medicine Database (PubMed), Medical Literature Analysis and Retrieval System Online (MEDLINE), Excerpta Medica Database (EMBASE), Cumulative Index to Nursing and Allied Health Literature (CINAHL), EBSCO, Web of science, Database of Abstracts of Reviews of Effects (DARE), Cochrane Database of Systematic Review, and Physiotherapy Evidence Database (PEDro). All relevant literature from the abovementioned databases will be retrieved for each research question. Table 2 shows the search terms including the Boolean operators that will be used for each research question in the PubMed database. A literature search will be conducted by a single reviewer using relevant subject headings, and keywords and modifications will be done based on the databases searched. All the articles will be retrieved and exported to EndNote software. As a first step, a single reviewer will de-duplicate in EndNote. The research assistant of the study will maintain a search log recording, search strategies, and search terms including Boolean operators that will be used for each database and for each research question, number of articles retrieved from each database, number of duplicates, and number of articles that will be archived at each phase of review.

**Table 2:** Search terms used for each research questions (PubMed)

Research questions	Search terms
Safely mobilizing patients with multiple myeloma in an acute care setting	((((("Multiple Myeloma" OR Myeloma OR "Plasma Cell Myeloma" OR "Haematological Cancer" OR "Bone Lesion*")) AND ("acute care" OR hospital OR Inpatient))) AND ("early mobili*" OR Mobili* OR "Early ambulat*" or ambulat*
Effective pain management in patients with multiple myeloma	(((("Multiple Myeloma" OR Myeloma OR "Plasma Cell Myeloma" OR "Haematological Cancer" OR "Bone Lesion*"))) AND (Pain OR "Pain Manag*" OR "Cancer Pain"))
Physiotherapy (PT) management in patients with multiple myeloma receiving chemotherapy	(((("Multiple Myeloma" OR Myeloma OR "Plasma Cell Myeloma" OR "Haematological Cancer" OR "Bone Lesion*")) AND (Physiotherapy OR "physical therapy" OR ambulat* OR mobili* OR "functional mobility training" OR exercis* OR "strength training" OR "endurance training" OR "cardio?pulmonary exercis*" OR "aerobic exercis*" OR "anaerobic exercis*" OR "high intensity exercis*" OR "low intensity exercis*" OR "home based exercis*" OR "multi-modal exercis*" OR "flexibility exercis*" OR "physical activity")) AND (chemotherap* OR "high-dose chemotherap*"))
PT management in patients with multiple myeloma receiving radiation	(((("Multiple Myeloma" OR Myeloma OR "Plasma Cell Myeloma" OR "Haematological Cancer" OR "Bone Lesion*")) AND (Physiotherapy OR "physical therapy" OR ambulat* OR mobili* OR "functional mobility training" OR exercis* OR "strength training" OR "endurance training" OR "cardio?pulmonary exercis*" OR "aerobic exercis*" OR "anaerobic exercis*" OR "high intensity exercis*" OR "low intensity exercis*" OR "home based exercis*" OR "multi-modal exercis*" OR "flexibility exercis*" OR "physical activity")) AND (Radiation* OR “Radiation therapy”))
PT management in patients with multiple myeloma receiving a stem cell transplantation	(((("Multiple Myeloma" OR Myeloma OR "Plasma Cell Myeloma" OR "Haematological Cancer" OR "Bone Lesion*")) AND (Physiotherapy OR "physical therapy" OR ambulat* OR mobili* OR "functional mobility training" OR exercis* OR "strength training" OR "endurance training" OR "cardio?pulmonary exercis*" OR "aerobic exercis*" OR "anaerobic exercis*" OR "high intensity exercis*" OR "low intensity exercis*" OR "home based exercis*" OR "multi-modal exercis*" OR "flexibility exercis*" OR "physical activity")) AND (“stem cell transplant*” OR “h?ematopoietic stem cell transplant*” OR “bone marrow transplant*”))
PT management in patients with multiple myeloma receiving a stem cell transplantation	(((("Multiple Myeloma" OR Myeloma OR "Plasma Cell Myeloma" OR "Haematological Cancer" OR "Bone Lesions")) AND (Physiotherapy OR "physical therapy" OR ambulat* OR mobili* OR "functional mobility training" OR exercis* OR "strength training" OR "endurance training" OR "cardio?pulmonary exercis*" OR "aerobic exercis*" OR "anaerobic exercis*" OR "high intensity exercis*" OR "low intensity exercis*" OR "home based exercis*" OR "multi-modal exercis*" OR "flexibility exercis*" OR "physical activity")) AND (“stem cell transplant*” OR “hematopoietic stem cell transplant*” OR “bone marrow transplant*”))
Management of fatigue and muscle weakness in patients with multiple myeloma	((("Multiple Myeloma" OR Myeloma OR "Plasma Cell Myeloma" OR "Haematological Cancer" OR "Bone Lesion*")) AND ("fatigue manag*" OR "cancer related fatigue" AND ("muscle weakness" OR "cancer related muscle weakness" OR "muscle weakness manag*"))
Management of fatigue and muscle weakness in patients with multiple myeloma	(("Multiple Myeloma" OR Myeloma OR "Plasma Cell Myeloma" OR "Haematological Cancer" OR "Bone Lesion*")) AND ("fatigue manag*" OR "cancer related fatigue" OR "muscle weakness" OR "cancer related muscle weakness" OR "muscle weakness manag*")

### Screening and selection of studies

Steps recommended by Pai et al. [23] will be followed in screening and selecting the studies. Each article in the EndNote software will be screened by two independent reviewers. In particular, the titles and abstracts of each article will be screened and include articles that meet the above described inclusion criteria and exclude all articles that are deemed irrelevant. The reviewers will meet and resolve any disagreements. Articles will be included if both the reviewers are in agreement. In case of disagreement, a third reviewer will be involved in the decision-making. All articles identified in the phase I screening process by mutual consent will be included in the phase II screening. In the phase II screening, full texts of all the identified articles in phase I screening will be thoroughly read and screened for the inclusion criteria by two independent reviewers. Articles considered eligible after full-text view by mutual consent will be included in the final analysis. The research assistant will continue to keep track of the number of articles and the rationale of excluding the articles at each of the screening phases. The research assistant will develop a flow diagram to report the number of articles included and excluded and the selection process as per the PRISMA guidelines.

### Data extraction and management

Two independent reviewers will extract the data from all the articles that are included in the final selection phase using a standardized data extraction form. The standardized data extraction form will be customized from the data extraction and assessment template proposed by “The Cochrane Public Health Group” [24]. Data extracted by mutual consent will be included for the final review. A third reviewer will assist in the decision-making upon disagreement between the two primary reviewers. The data extraction form will be pilot tested with two articles and will be revised based on suggestions by the team members. The following data will be extracted from each article: (1) general study details: Title, authors, source, country of study, publication type, and year of publication; (2) study eligibility: type of study, participants characteristics including, number of participants, age, gender, diagnosis, type of cancer treatment, stage of cancer, inclusion/exclusion criteria, setting, methods including design/allocation, blinding, sampling, loss to follow-up, recruitment rates, retention rates, and adherence rates, intervention characteristics including type of exercise/physical activity, types of outcome measures including self-reported outcomes, objective outcomes; (3) study details: aim of study, aim of intervention, details of intervention including, setting, strategies used, frequency, intensity, duration, program length, provider, co-interventions, subgroups, details of outcomes, and results of the study. The data extraction will be documented in a Microsoft Excel spreadsheet.

### Critical appraisal process

Each article will be critically appraised using standardized critical appraisal tools including the PEDro scale, the Methodological Index for Non-Randomized Studies (MINORS), the Critical Appraisal Skills Programme (CASP) checklists [25], the Measurement Tool to Assess Systematic Reviews (AMSTAR) [26–28], and the Appraisal of Guidelines for Research and Evaluation II (AGREE II) tool [29] to ascertain adequate quality and appropriateness of the evidence to be used in guideline development. The above-described tools are recommended by the APTA [16] and have been used in guideline development by other groups [17, 30, 31]. Critical appraisals will be performed by two independent reviewers. The two reviewers will undergo training and will practice the critical appraisal tools before doing the actual critical appraisals. The scores between the two reviewers will be compared, and discrepancies will be resolved. A third reviewer will be involved upon disagreement.

Scientific conclusions will be drawn and recommendations will be made based on the critical appraisals, the guideline development team’s (team with expertise on oncology, physiotherapy, exercise specialists, clinical psychology, teaching, and research) judgment about the overall quality of the studies and consensus from the expert group.

### Levels of evidence

The levels of evidence for each article will be determined based on the recommendations of Kaplan and colleagues [32] and critical appraisal scores. Evidence obtained from high-quality studies (≥ 50% critical appraisal scores) will be assigned “level I” and evidence obtained from lesser quality studies (< 50% critical appraisal scores) will be assigned “level II.” Studies with unacceptable quality will be excluded from consideration in the guideline. Table 3 shows the description of the levels of evidence that will be used for determining levels of evidence of the articles.

**Table 3:** Levels of evidence

Level	Criteria
I	Evidence obtained from high-quality diagnostic studies, prognostic or prospective studies, cohort studies or randomized controlled trials, meta analyses or systematic reviews (critical appraisal score *>* 50% of criteria).
II	Evidence obtained from lesser-quality diagnostic studies, prognostic or prospective studies, cohort studies or randomized controlled trials, meta analyses or systematic reviews (eg, weaker diagnostic criteria and reference standards, improper randomization, no blinding, *<*80% follow-up) (critical appraisal score *<*50% of criteria).
III	Case-controlled studies or retrospective studies
IV	Case studies and case series
V	Expert opinion

Reprinted from Kaplan SL, Coulter C, Fetters L. Developing evidence-based physical therapy clinical practice guidelines. Pediatr Phys Ther. 2013;25:257–270, with permission of Wolters Kluwer Health Inc.

### Grades of action statements

The Grading of Recommendations, Assessment, Development and Evaluation (GRADE) will be used to determine the strength of evidence [33]. See Table 4 for the criteria for the grades of recommendations for action statements. The guideline development team will use their judgment and weigh the quality of the collective evidence and potential benefit against potential harm for assigning grades to the action statements as recommended by the APTA [16].

**Table 4:** Definition of Grades of Recommendation for action statements

Grade	Recommendation	Quality of Evidence
A	Strong	A preponderance of level I studies, but least 1 level I study directly on the topic support the recommendation.
B	Moderate	A preponderance of level II studies but at least 1 level II study directly on topic support the recommendation.
C	Weak	A single level II study at less than 25% critical appraisal score or a preponderance of level III and IV studies, including statements of consensus by content experts support the recommendation.
D	Theoretical/foundational	A preponderance of evidence from animal or cadaver studies, from conceptual/theoretical models/principles, or from basic science/bench research, or published expert opinion in peer-reviewed journals supports the recommendation.
P	Best practice	Recommended practice based on current clinical practice norms, exceptional situations where validating studies have not or cannot be performed and there is a clear benefit, harm, or cost, and/or the clinical experience of the guideline development group.
R	Research	There is an absence of research on the topic, or higher-quality studies conducted on the topic disagree with respect to their conclusions. The recommendation is based on these conflicting or absent studies.

Reprinted from Kaplan SL, Coulter C, Fetters L. Developing evidence-based physical therapy clinical practice guidelines. Pediatr Phys Ther. 2013;25:257–270, with permission of Wolters Kluwer Health Inc.

### Linking levels of evidence and grades of action statements

The following terms will be used in describing levels of obligation as recommended by the APTA ([16], pg. 32). The term *must* will be used if the evidence supports a strong recommendation (grade A) and harm might occur if the action is not followed. The term *should* will be used if the evidence supports a strong (grade A) or moderate (grade B) recommendation. The terms *may* or *could* will be used if the evidence is weak (grade C).

### Creating action statements

Recommendations will be made in the form of action statements. Brief, precise, quality-driven action-oriented statements will be created using a BRIDGE-Wiz software as recommended by the APTA. The action statements will be described in terms of *Who*, *When* (*under what specific conditions*), *Must*, *Should*, *or May* (*the level of obligation*), *Do what* (*precise action*), and *to Whom*. Action statements will be supplemented by action statement profiles listing aggregate decisions made by the guideline development team and consensus process. The action statement profiles will include the following headings: aggregate evidence quality, level of confidence in evidence, benefits, risks, harm, costs, benefit-harm assessment, value judgments, intentional vagueness, role of patient preferences, exceptions, policy level, and differences of opinion [16, 34].

### Consensus process

Five physiotherapists across Canada experienced with the care of patients with MM will independently review the contents of the guidelines and provide feedback. Aggregate level of evidence will be assigned after the group reaches consensus on the evidence supporting each key action statement. The guidelines will be revised till 80% consensus is achieved on each of the statements and profiles. Action statements that do not reach 80% consensus after three rounds of consensus process will be removed from the guideline document.

### Internal review

Three independent reviewers will evaluate the guideline using AGREE II. It is a reliable and valid instrument used to assess the quality of clinical practice guidelines. AGREE II consists of 23 items clustered under 6 domains (scope and purpose, stakeholder involvement, rigor of development, clarity of presentation, applicability, and editorial independence) developed to assess the quality of the guidelines. Each item in the questionnaire is evaluated on a 7-point scale where score 1 refers to strong disagreement and score 7 refers to strong agreement.

### External review

The guideline will undergo two external reviews. The guideline will be sent to CPA for feedback from physiotherapists. The guideline will also be reviewed by a methodological expert from Evidence-Based Care - Cancer Care Ontario. The draft guideline will be posted on the Health Sciences North (HSN) website for public comment.

### Strengths and limitation of this study

This study will employ validated methodologies such as PICO guidelines, systematic reviews, and consensus process. This study will also use standardized quality assessment tools for specific research designs. The literature search and data extraction processes will be done by independent reviewers thereby enhancing the validity of the study. Additionally, the review panel will consist of experts in patients with MM. Moreover, the formulated guidelines will also be evaluated using the AGREE II instrument, which adds to the validity of this study.

The following study limitations are noted. We acknowledge that this study might have significant result publication bias, meaning that this study might review articles showing significant results (effects) on earlier described research question, which tells nothing about possible ineffective interventions, as non-significant results are less likely to be published; however, this is beyond the control of any researcher. This study may be subjected to selection bias by not including relevant articles published in languages other than English. Reporting bias will be minimized by having two independent reviews and by having a third independent reviewer intervene when there are disagreements at each stage of data identification, data extraction, and critical appraisal.

### Clinical and research implications

The outcomes of the study are expected to benefit practitioners because it will provide clear evidence-based guidelines regarding best practices to administer physiotherapy treatment to patients with MM. The recommendations made from this study will be based on rigorous and validated methods that will assist in minimizing potential harms and improve the quality of care. The patients will benefit from receiving the best possible treatment that is tailored to their unique set of health concerns. Rather than using a “one-size fits all” approach to PT treatment, the study will focus specifically on patients that have MM and will seek to determine what specific PT treatment options are best suited for this population. The benefits could be measured qualitatively by requesting input from practitioners regarding the guidelines (clarity and ease of implementation). The benefits to the patients could be assessed in a follow-up study comparing standard PT care compared to the MM-tailored PT intervention program. The findings of this study will also provide a comprehensive platform for future research directions by identifying gaps in the literature.

## Additional file

Additional file 1: PRISMA-P checklist. (DOCX 26 kb)

## Abbreviations

AMSTAR: A Measurement Tool for the Assessment of Multiple Systematic Reviews
APTA: American Physical Therapy Association
CPA: Canadian Physiotherapy Association
GRADE: Grading of Recommendations Assessment, Development and Evaluation
MINORS: Methodological Index for Non-Randomized Studies
MM: Multiple myeloma
PEDro: Physiotherapy Evidence Database
PICO: Population, intervention, control, and outcomes
PRISMA-P: Preferred Reporting Items for Systematic Reviews and Meta-Analysis Protocols
PROSPERO: International Prospective Register of Ongoing Systematic Reviews
PT: Physiotherapy/physical therapy
